# The Development of Quality Control Genotyping Approaches: A Case Study Using Elite Maize Lines

**DOI:** 10.1371/journal.pone.0157236

**Published:** 2016-06-09

**Authors:** Jiafa Chen, Cristian Zavala, Noemi Ortega, Cesar Petroli, Jorge Franco, Juan Burgueño, Denise E. Costich, Sarah J. Hearne

**Affiliations:** 1 International Maize and Wheat Improvement Center (CIMMYT); Texcoco; Edo. De Mexico; Mexico CP 56237; 2 Departamento de Biometría, Universidad de la Republica, Paysandú; Uruguay CP 60000; Jawaharlal Nehru University, INDIA

## Abstract

Quality control (QC) of germplasm identity and purity is a critical component of breeding and conservation activities. SNP genotyping technologies and increased availability of markers provide the opportunity to employ genotyping as a low-cost and robust component of this QC. In the public sector available low-cost SNP QC genotyping methods have been developed from a very limited panel of markers of 1,000 to 1,500 markers without broad selection of the most informative SNPs. Selection of optimal SNPs and definition of appropriate germplasm sampling in addition to platform section impact on logistical and resource-use considerations for breeding and conservation applications when mainstreaming QC. In order to address these issues, we evaluated the selection and use of SNPs for QC applications from large DArTSeq data sets generated from CIMMYT maize inbred lines (CMLs). Two QC genotyping strategies were developed, the first is a “rapid QC”, employing a small number of SNPs to identify potential mislabeling of seed packages or plots, the second is a “broad QC”, employing a larger number of SNP, used to identify each germplasm entry and to measure heterogeneity. The optimal marker selection strategies combined the selection of markers with high minor allele frequency, sampling of clustered SNP in proportion to marker cluster distance and selecting markers that maintain a uniform genomic distribution. The rapid and broad QC SNP panels selected using this approach were further validated using blind test assessments of related re-generation samples. The influence of sampling within each line was evaluated. Sampling 192 individuals would result in close to 100% possibility of detecting a 5% contamination in the entry, and approximately a 98% probability to detect a 2% contamination of the line. These results provide a framework for the establishment of QC genotyping. A comparison of financial and time costs for use of these approaches across different platforms is discussed providing a framework for institutions involved in maize conservation and breeding to assess the resource use effectiveness of QC genotyping. Application of these research findings, in combination with existing QC approaches, will ensure the regeneration, distribution and use in breeding of true to type inbred germplasm. These findings also provide an effective approach to optimize SNP selection for QC genotyping in other species.

## Introduction

The CIMMYT (International Maize and Wheat Improvement Center) Maize Lines (CMLs) are a set of 577 elite inbred lines, which have been developed over the last 25 years. The CMLs represent one of the most widely distributed sources of publically generated elite lines, which are freely available to both public and private sector breeders, research and growers, worldwide. Distributed under the Standard Material Transfer Agreement (SMTA) of the International Treaty on Plant Genetic Resources for Food and Agriculture (ITPGRFA), these lines have become the most important public tropical maize germplasm source globally [1]. Since 2005 the conservation, regeneration and distribution of CMLs has been the responsibility of the CIMMYT Germplasm Bank (CGB). The CMLs are the most requested accessions that the CGB holds and as such are subject to the most frequent regenerationsTherefore, during which, the possibility for contamination is always present. In order for CGB to maintain these materials as true to type, pure and stable lines, we determined that a stringent QC genotyping system should be implemented.

Control and understanding of germplasm identity and purity are two fundamentals of germplasm management, be it for breeding or conservation [2–4]. Inability to rapidly and cost effectively assess these characteristics restricts the accuracy and precision of breeding and may have an impact on the integrity and usefulness of genebanks. Molecular markers have been widely used to understand the genetic relationships among species, populations and individuals, and also to identify the causal loci for specific traits [5–10]. They have several advantages as compared to morphological markers, including high polymorphism, high-throughput detection methods, and they are unaffected by environmental conditions or the physiological stage of the plant [11,12]. Germplasm QC genotyping based on molecular markers has long been proposed as an effective component of QC [13–15].

A Single nucleotide polymorphism (SNP) is a single base difference within otherwise identical sequences of DNA found at one position in the genome. In recent years, the adoption of next generation DNA sequencing technologies has significantly increased the number of SNP markers available for use in crops [16,17]. Genotyping by sequencing (GbS) and other sequence based technologies like DArTSeq have evolved as one of the principal methods for next generation sequence based genotyping. A variety of approaches for sequence based genotyping can be employed, each tailored for different applications. For example the GbS methods developed and used at Cornell University [18] generate a very high density of markers (>800,000 SNP) with relatively low coverage (depth of sequencing at particular loci, 0.5X) and a missing data rate of more than 50%. These data are very effectively used with studies employing structured populations where imputation is deployed with high accuracy to address missing data and generate heterozygote calls [19]. This GbS approach is critical in studies involving genome wide association where high marker density is a requisite. In contrast, other approaches currently used in maize, like DArTseq [20], generate a lower density of markers (50,000 to 350,000 SNP) but have much higher coverage and lower levels of missing data (20% and lower) in comparison with Cornell implementations of GbS for maize. The additional identification of presence absence variation (a further 40,000 to 200,000 markers) and ability to directly score heterozygotes/heterogeneous samples with this lower density approaches has broad application in diversity related and genomic selection applications. Given the high numbers of markers available, relatively low cost and high speed of detection, SNPs are currently considered to be the optimal marker type for QC genotyping in crops [21–24].

A number of studies have addressed various aspects of QC genotyping in maize. Semagn *et al*. used a total of 28 maize inbred lines to study genetic identity among different seed sources, genotyping them with 532 and 1,065 SNPs using the KASP and Golden-Gate platforms, respectively [14]. The results showed the proportion of alleles differing between seed sources of the same inbred line varied from 0.1 to 42.3%. Seed sources exhibiting high levels of genetic distance were miss-labeled, while those with lower levels of difference were considered to be contaminated or still segregating. The authors recommended using a subset of 50–100 SNPs for routine and low-cost QC genotyping, which was verified in a different set of double haploid and inbred lines. In a second study, Ertiro et al., used 191 KASP and 257,268 imputed Cornell GbS markers to evaluate the level of genetic purity (defined as homogeneity across markers) and identity among two to nine seed sources of 16 inbred lines [25]. The results showed genetic purity within each seed source varied from 49 to 100% for KASP and from 74 to 100% for GbS. There was high discrepancy both in genetic purity and identity by the origin of the seed sources (institutions) irrespective of the type of genotyping platform and number of markers used for analyses (100 and 191 KASP compared with all GbS). The correlation between the KASP and GbS platform was 0.88 for purity and 0.93 for identity, suggesting that a smaller subset of high quality markers are sufficient for QC analysis. These studies provide a good framework regarding QC genotyping but they do not address the best approach to select a defined sub-set of markers, optimized to facilitate QC genotyping, nor do they address in any way the question of sampling of germplasm for QC genotyping (both studies involved the genotyping of one bulk of DNA from 10 plants per entry).

A number of factors can impact the cost of generation of genotypic data for QC purposes. The number of markers employed for QC genotyping can influence the cost per sample depending to some extent on the genotyping platform selected; single plex systems like KASP being influenced much more than array type and sequence based platforms. In addition, the number of sub-samples used for QC genotyping increases the cost per sample in a platform independent manner. Different stages of breeding and germplasm bank activities have varied tolerance to cost and accuracy of genotyping. For example, the development of new breeding populations is a stage where breeders require higher accuracy of data (reflecting the importance of inbreeding status in the maize pedigree breeding process). In addition, due to lower numbers of breeding starts compared with subsequent sample volumes occurring in the breeding discovery and selection processes, there is greater cost benefit to increase marker number and number of samples genotyped to ensure accuracy of data and data interpretation at this stage. Optimization of marker density and sampling strategy are key factors in determining genotyping costs, these need to be balanced with the required function/purpose of the genotypic data. Generation of and/or selection of sub-sets of high quality markers for QC is an important decision for breeding and germplasm conservation activities. Here we define an approach for selecting markers for two contrasting QC genotyping approaches. The first is a “rapid QC”, employing a limited number of SNPs to cost effectively identify the mislabeling of seed packages or plots used to regenerate accessions in the field. The second is “broad QC”, employing a larger number of SNPs, to identify each line, and to estimate heterogeneity and, if individual plants are sampled, heterozygosity. The costs and accuracy tradeoff in the application of these methods is also addressed across common platforms to provide breeders with information that they can use when determining what platforms and levels of resolution they require from genotypic data. The effects of a number of parameters on QC genotyping were studied: (1) the methods used to select markers; (2) the number of markers needed for broad QC versus rapid QC genotyping; (3) the optimization of marker subsets for both QC genotyping approaches; and (4) the influence of sample size on accurate identification of each line assessed.

## Materials and Methods

### Plant Material and DNA extraction

Seed samples and the passport data of all of the 561 CMLs (available in 2014) were obtained from the CGB [To order seed, go to: http://www.cimmyt.org/seed-request. To find more information about CMLs, go to: http://hdl.handle.net/11529/10246]. These seed samples were delivered from the original seed lot provided to the CGB in 2005. In addition, seed samples of 22 CMLs from multiple regenerations were also obtained (S1 Table). Seedlings were established in pots in screenhouses at the CIMMYT Headquarters farm in El Batán, Texcoco, Mexico. Leaf tissue from 12 plants per sample were harvested, the tissue was lyophilized, and the DNA was extracted from a composite of equal area (28 mm^2^) of leaf tissue from each individual plant using a modified CTAB method [26]. DNA was quantified, diluted to equal concentration (200 ng/ul) and submitted for genotyping by DArTseq method to Diversity Arrays Technology (DArT) (www.diversityarrays.com/).

### Genotyping

A High-throughput genotyping method was conducted in 96 plex using DArTseqtechnology [20,27]. A genomic representation of the set of samples was generated by digesting the genomic DNA with a combination of two restriction enzymes, PstI (CTGCAG) and HpaII (CCGG), and ligating barcoded adapters to identify each sample. For each 96 well plate, 16% of the samples were replicated to assess reproducibility. Equimolar amounts of amplification products from each sample were pooled by plate and amplified by c-Bot (Illumina) bridge PCR, followed by fragment sequencing on Illumina Hiseq 2500 (www.illumina.com). SNPs were called using the DArTsoft analytical pipeline (http://www.diversityarrays.com/software.html#dartsoft). Sequence analysis was conducted to align reads with the sequence tag based maize meta-genome representation. A total of 88,600 unimputed SNPs were successfully called within the CML dataset. Data derived from this analysis included both SNP calls and the number of sequence reads per allele.

### Data analysis

The characteristics of each SNP marker, such as allele frequency [28], heterogeneity, percentage of missing and polymorphic information content (PIC) [29,30], were computed using the R statistical package [31]. The coverage of each SNP was calculated as the average number of reads per allele. The allele sharing distance matrix between all pairs of individuals was constructed as described by Gao and Starmer [32,33], and it was used to calculate the CML cluster and the Principal Coordinate Analysis for CMLs. The maximum allele sharing distance is 2, and the minimum distance is 0 (Allele sharing distance is two times the dissimilarity). The similarity between two samples was calculated by the total number of identical alleles divided by the total number of non-missing alleles, which was used in blind test to identify the samples. The maximum similarity is 1, and the minimum is 0. The r^2^ of LD (linkage disequilibrium) between two markers was calculated in R, using the method described by Devlin [34]. The “complete” method of hclust function in R was used to cluster the populations and generate the tree file, using the allele sharing distance matrix. The tree picture was generated by FigTree v1.4.0 (http://tree.bio.ed.ac.uk/software/figtree/). Principal Coordinate Analysis (PCoA) of all CMLs was carried out using cmdscale function in R, and the PCoAs and categorical data were plotted by CurlyWhirly v1.15 (http://ics.hutton.ac.uk/curlywhirly/). Principal component analysis (PCA) and K-means were used to group the SNP markers using the prcomp and kmeans functions in R [35,36]. Some CML are trait conversions of earlier materials. Separation of these closely related converted lines from their CML parents is most easily achieved through use of markers specifically associated with the introgressed trait. In order to identify the trait-specific markers, trait values for the conversion characteristics QPM (quality protein maize) and imidazolinone resistance were evaluated using a numeric classification of each trait; 1 for presence of trait and 0 absence (data available in http://hdl.handle.net/11529/10246). The allele case control test was then performed using PROC CASECONTROL function in SAS/Genetics V12.3 to evaluate all markers’ p-value for QPM and imidazolinone resistance [37,38]. The most significant marker from the analysis was used as a trait-specific marker.

To determine the influence of sample number on the accuracy of detecting off-types in QC genotyping, we calculated the probability of finding at least one off-type from different sample sizes for a set of defined levels of off-types (0.001, 0.01, 0.02, 0.05 and 0.1). The model assumed that the marker detection power was hundred percent (the markers used could detect all off-types in the available sub-samples). Using a binomial distribution, probability of detection at least one off-type (P) was calculated using the following formula: P = 1 –(1-p)^n^, where n is the sample size and p is the percentage of off-type expressed as a probability. Bayesian estimation of the proportion of off-types in the population was used with a Beta prior distribution by the “binom” library in R (R-project) [31]. To estimate the percentage of off-types in populations based on known percentage of off-types in samples, the proportion of off-types in the populations were determined by the 0.95 percentile of the posterior distribution of the parameter p for different sample sizes and number of off-types in the samples [39].

## Results

### SNP profiles

**Fig 1:**
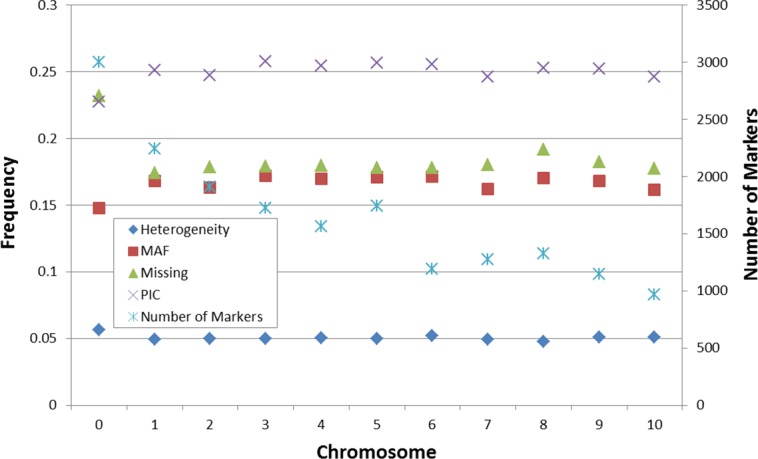
Summary of the heterogeneity, minor allele frequency (MAF) and polymorphic information content (PIC) of 18,082 selected SNPs. Chromosome assignments are indicated; where no BLAST position was available, the chromosome is designated as “0”; The heterogeneity, MAF, percentage of Missing value, PIC was shown in left y-axis, the number of marker for each chromosome was shown in right y-axis.

A total of 88,600 SNP markers were obtained from the DArTseq platform. SNPs were initially filtered to remove SNPs with missing rate >40%, SNPs with minor allele frequency (MAF) < 5% and heterogeneity >10%, resulting in 18,082 markers used for subsequent analyses (S2 Table), these genotypic data are available in: http://hdl.handle.net/11529/10431. Per chromosome, the average marker heterogeneity was approximately 0.05, the proportion of missing values was close to 0.18, and the minor allele frequency (MAF) and polymorphic information content (PIC) were around 0.16 and 0.25 respectively (Fig 1). The SNP mutation type analysis indicated that 60.7% of the SNP mutations belong to A/G, T/C type; the remaining mutations belong to A/C, T/G type (19.5%), and A/T, C/G type (19.8%).

PCoA of CMLs using selected SNPs produced clear separation of groups based on adaptation (S1 Fig) and grain color (S2 Fig). Hierarchical clustering showed clear definition by germplasm source, with CMLs clustering into anticipated population-based groups (S3 Fig). These results were as expected and confirm that the genotypic data gave clear and accurate representations of the genetic space of the CMLs. All paired CMLs showed a mean polymorphism of 27.5%, similar to the results obtained using other genotyping platforms, i.e., KASP and Illumina Golden-Gate [14].

### Analysis of methods to enable selection of markers for QC

**Fig 2:**
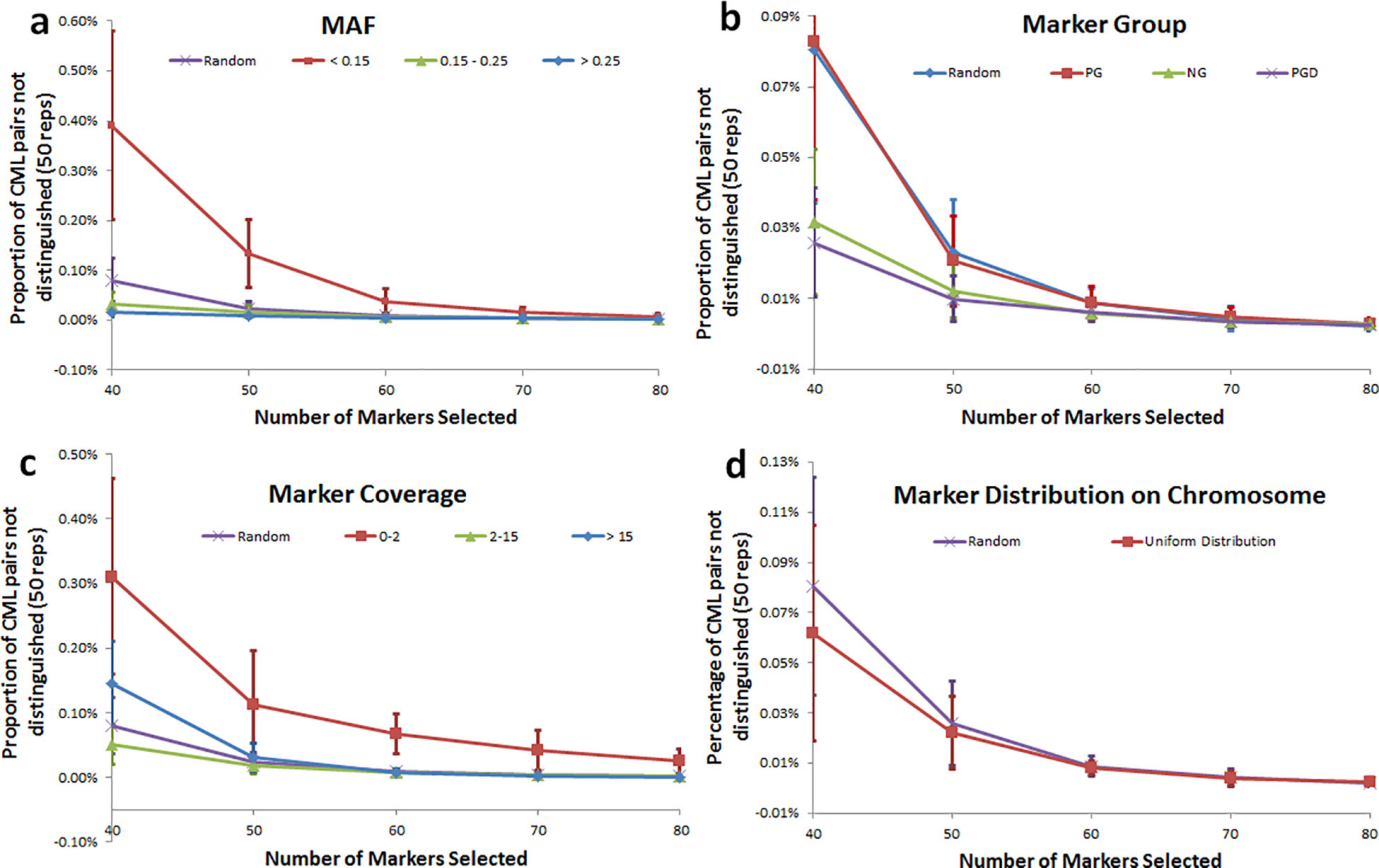
Analysis of methods to enable selection of markers for QC genotyping. a: MAF effect. Random selection (Random); MAF < 0.15 (0.05–0.15); MAF between 0.15 and 0.25 (0.15–0.25); MAF > 0.25 (>0.25); b: Marker Group effect on select marker for QC. Random selection (Random); Same percentage of marker from each marker group (PG); same number of markers selected from each group (NG); Keep the proportion of each group distance (PGD). c: Marker coverage effect. Random selection (Random); Coverage < 2 (0–2); Coverage between 2 and 15 (2–15); Coverage >15 (>15). d: Marker distribution effect. Standard error bars are shown.

In order to select the most informative markers for QC, a number of different marker parameters were evaluated, including MAF, marker group, coverage, and chromosome position (Fig 2). The markers were separated into three groups with similar number of SNPs by MAF: 1) MAF < 0.15; 2) MAF between 0.15 and 0.25; 3) MAF > 0.25. A random selection of all MAF was also evaluated as a control (Fig 2A). Comparison of the effect of different SNP MAF on proportion of CML pairs not distinguished showed the higher MAF SNP had improved efficiency to distinguish CMLs from one another. Selection of higher MAF would therefore enhance the effectiveness of QC genotyping applications (Fig 2A).

In order to evaluate the effect of marker group, markers were clustered into groups using PCA and K-means methods [35,36] (S4 Fig). Random selection of markers from each of the five groups defined was conducted based on a series of selection criteria; 1) Complete random selection without marker group information (Random); 2) Equal proportions of markers selected from each marker group (PG); 3) Equal number of markers from each group (NG); 4) A number of markers selected from each group proportional to average group distance (PGD). The effects of the four selection methods were studied based on 50 random samplings. There was no difference between Random and PG selection with respect to the ability to distinguish of CML pairs. NG and PGD method produced similar results, however, the PGD method produced a little better separation of CMLs at a lower marker number (Fig 2B).

Assessment of marker coverage (sequencing depth at a marker locus) showed that coverage was proportional to heterogeneity and inversely proportional to missing data measures, as would be expected. The increase in measured heterogeneity with increasing coverage reflects the improved probability of sampling multiple variants at a locus if they exist (S5 Fig), while the probability of sampling any allele at a locus increases with coverage, hence influenced by technically missing data. Above a coverage threshold of 2 there was less variation in both missing data and heterogeneity as coverage was increased. Based on this result and number of makers at different coverage threshold, SNPs were separated into three groups for analysis of coverage effect on marker selection for QC: 1) Coverage < 2; 2) Coverage between 2 and 15; 3) Coverage > 15. A random selection of markers was used for comparison. Marker coverage between 2 and 15 had the highest efficiency to distinguish different CML, indicating that this range of coverage was optimal for CML differentiation (Fig 2C).

Marker distribution effects were studied by comparing randomly distributed markers versus markers with uniform distribute across chromosomes. Uniform marker selection demonstrated a better separation of CMLs than random selection (Fig 2D).

**Fig 3:**
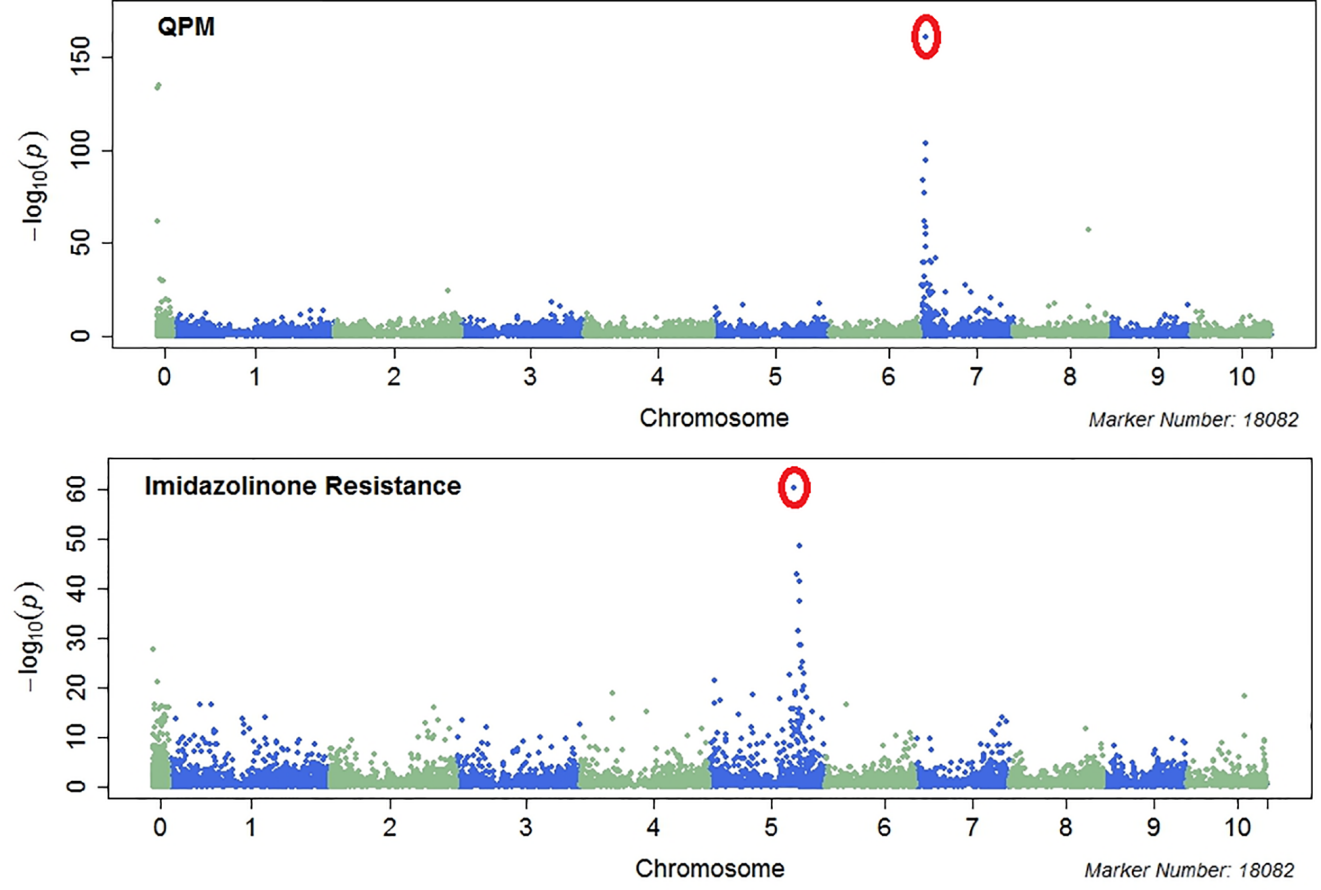
Association analysis indentifies QPM and imidazolinone resistance markers.

In the CML panel, there were two simple traits, quality protein maize (QPM), controlled by the o2 gene [40,41] and native imidazolinone resistance (IR) controlled by the als2 gene [42], that have been introgressed via backcrossing to convert existing CMLs. Conversions result in new lines with novel features which can be difficult to distinguish visually from the recurrent CML parents in a rapid manner without equipment. It was therefore important to include trait-specific markers to enable differentiation of these converted lines from their recurrent parents. For example, CML503 is the QPM converted version of CML264, and CML523 is the IR converted version of CML445 (S3 Table). Using Genome-Wide Association Study (GWAS) to assess introgression, two trait-specific markers were defined for application in CML QC genotyping (Fig 3).

Based on the analysis of the effects of marker characteristics on the accuracy of QC genotyping for CMLs, the following marker selection rules were implemented to define markers for evaluation: 1) Marker coverage between 2 and 15; 2) Missing value less than 20%; 3) Remove SNPs without chromosome information; 4) Marker heterogeneity less than 6%; 5) Inclusion of QPM and IR specific markers. Using these stringent marker selection criteria, 193 markers across the whole genome were chosen for re-sampling analysis.

**Fig 4:**
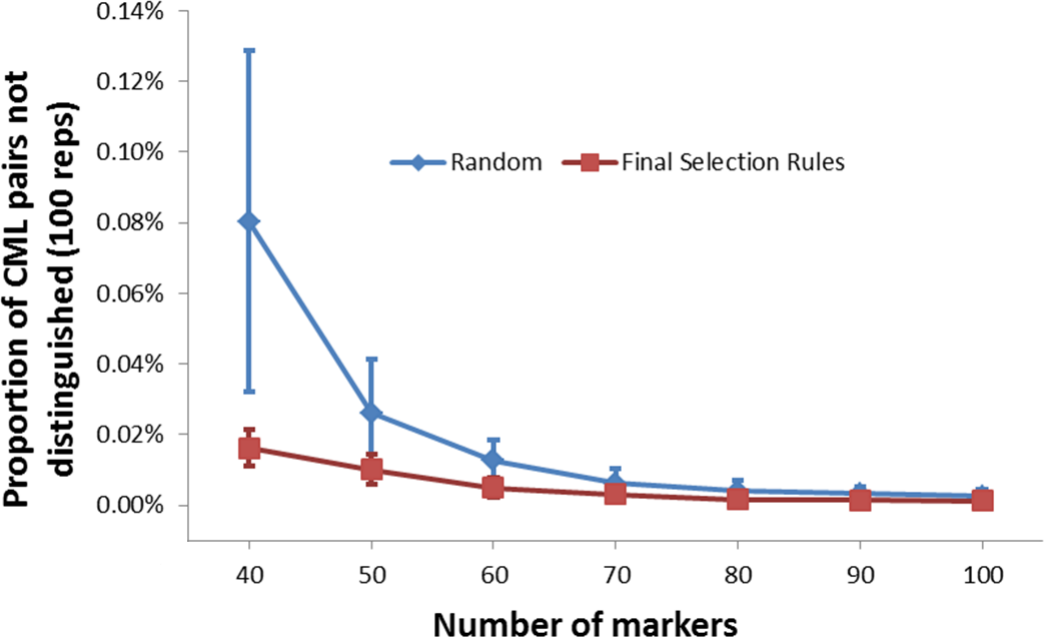
Comparison of the effect of the final marker selection rules versus random marker selection on the proportion of CML pairs not distinguished from one another.

A series of between 40 to 100 markers were randomly selected from the defined panel of markers, based on uniform chromosome distribution and PGD. This selection, compared with random selection, provided a better definition of CMLs (Fig 4). Analysis also indicated all CMLs could be distinguished very well using 80 markers, suggesting that for systems based on single plex assays, where increasing markers results in increasing cost, 80 selected markers would provide sufficient information for CML broad QC genotyping, given the current makeup of the CML panel. For chip or sequence based SNP detection systems, where marker number is not a major cost constraint, it would be highly desirable to increase the marker number in order to improve accuracy and enable the addition of further unknown lines.

### Definition of SNPs for QC

**Fig 5:**
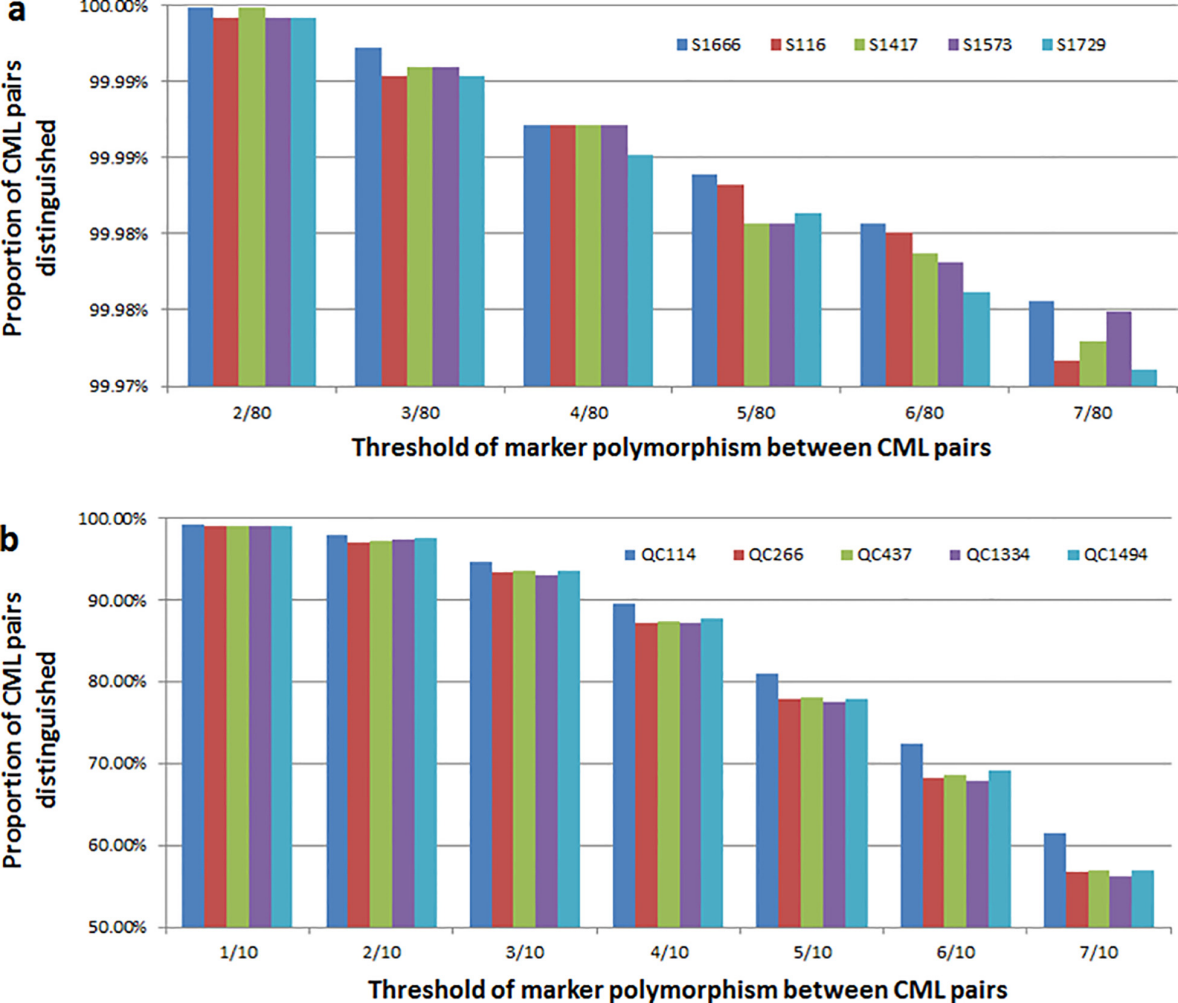
Comparison of five subsets of SNPs for broad QC and rapid QC. a: Five subsets of 80 SNPs for broad QC genotyping; b: Five subsets of 10 SNPs for rapid QC genotyping.

In order to define the best 80 SNPs for broad QC, replicated randomized re-sampling of SNPs from the panel of 193 SNPs was conducted, including the QPM and IR trait-specific markers (2,000 reps). Of the 2,000 re-sampled sets, five subsets could successfully distinguish all CMLs. In order to identify the best subset of the five, the ability of each subset to differentiate pair-wise CML comparisons was assessed (Fig 5A). Subset S1666 (S2 Dataset) showed the highest ability to distinguish CMLs and as such was considered the best subset for broad QC. For rapid QC genotyping, fewer than 80 markers would be desired by most breeding programs in situations where single plex marker systems were used (lower cost and faster data return). The separation of CMLs was evaluated using replicated random re-sampling (100 reps) of between 6 to 20 markers within subset S1666. Six markers could identify 90% to 94% of cases of miss-identity, while ten markers could identify error in 97% to 99% of cases (S6 Fig). Ten markers were considered sufficient for rapid QC since other features, such as seed color and plant morphology, could be used by breeders and genebank staff in combination with rapid QC analysis to detect miss-identity [43]. To define the best ten SNPs, random re-sampling of ten markers was conducted with 2,000 replications within the best broad QC subset (S1666). Five subsets of markers were identified which provided excellent differentiation of CMLs. Comparison of the five subsets indicated that subset QC114 provided the best differentiation of CML pairs (Fig 5B) (S2 Dataset).

### Validation of selected markers

CMLs are frequently requested by users both internal and external of CIMMYT, and therefore must be frequently re-generated for distribution. For 22 CMLs, a series of regenerations, including the original source, were genotyped using DArTSeq platform. The genotypic similarity of the 22 original CMLs ranges from 0.44 to 0.76 (S3 Dataset). Genotypic data from these entries were filtered, and the analysis was conducted using only the 80 selected markers from S1666. Using these markers, blind tests showed that, in all but one case, the most recent and intermediate regenerations of each of the CMLs were most similar in identity to the original CML (Table 1). One CML regeneration (CML233 re-generation 2) was identified as a different line, sharing high similarity to CML132 (0.95). It shared only 0.60 similarities to the original CML233 samples. This misalignment is likely due to a mislabeling of a sample during seed processing or DNA extraction. In the case of CML14, the four regenerations had highest similarities to the original CML14, but the similarity was markedly lower than other comparisons. Further analysis of the original CML14 sample revealed a high rate of heterogeneity (12.3%); this was lower in subsequent generations (1.7%). This result indicates that the original CML14 wasn’t as homozygous as expected for an inbred line, and it responded as expected to a phenotypic purification procedure conducted during regeneration. Analysis of the ten markers for rapid QC provided similar results (Table 2). Both results showed that 80 broad QC markers and 10 rapid QC markers worked very well, providing evidence of mislabeling of samples and potential issues with residual heterogeneity.

**Table 1:** Similarity between original CMLs and re-generation using 80 broad QC markers. Genotypic similarity is indicated in parentheses.

Line Name	Blind test identified the most similar original CML and their genetic similarity				
	re-generation 1	re-generation 2	re-generation 3	re-generation 4	re-generation 5
CML100	CML100(0.94)	CML100(0.97)	CML100(0.96)	CML100(0.93)	CML100(0.94)
CML106	CML106(0.98)	CML106(0.99)	CML106(0.98)	CML106(0.97)	CML106(0.99)
CML110	CML110(0.89)	CML110(0.89)	CML110(0.90)	CML110(0.90)	CML110(0.89)
CML126	CML126(0.96)	CML126(0.99)	CML126(0.99)	CML126(0.99)	-
CML131	CML131(0.99)	CML131(0.99)	CML131(1.00)	CML131(0.99)	CML131(0.99)
CML136	CML136(0.99)	CML136(0.99)	CML136(0.99)	CML136(1.00)	-
CML14	CML14(0.84)	CML14(0.86)	CML14(0.87)	CML14(0.85)	-
CML17	CML17(0.93)	CML17(0.95)	CML17(0.95)	CML17(0.93)	CML17(0.95)
CML178	CML178(0.97)	CML178(0.95)	CML178(0.95)	CML178(0.98)	CML178(0.94)
CML192	CML192(0.96)	CML192(0.98)	CML192(0.99)	CML192(0.96)	CML192(0.99)
CML193	CML193(0.95)	CML193(0.95)	CML193(0.89)	-	-
CML197	CML197(0.99)	CML197(1.00)	CML197(0.99)	CML197(1.00)	CML197(1.00)
CML233	CML233(0.94)	*CML132*(0.95)*	CML233(0.99)	-	-
CML236	CML236(0.94)	CML236(0.91)	CML236(0.93)	-	-
CML280	CML280(0.97)	CML280(0.99)	CML280(0.99)	CML280(0.98)	-
CML327	CML327(0.97)	CML327(0.99)	CML327(0.90)	CML327(0.96)	CML327(0.99)
CML362	CML362(0.97)	CML362(0.98)	CML362(0.97)	CML362(0.98)	-
CML364	CML364(0.99)	CML364(1.00)	CML364(1)	CML364(1.00)	-
CML390	CML390(0.97)	CML390(0.97)	CML390(0.97)	CML390(0.96)	CML390(0.97)
CML393	CML393(0.98)	CML393(0.99)	CML393(0.99)	CML393(1.00)	CML393(0.98)
CML435	CML435(0.96)	CML435(0.97)	CML435(0.99)	CML435(0.97)	-
CML82	CML82(0.96)	CML82(0.97)	CML82(0.96)	CML82(0.95)	-

* Re-generation 2 of CML233 has only 0.60 similarity to the original CML233 sample

**Table 2:** Similarity between original CMLs and different re-generation using 10 rapid QC markers. Genotypic similarity is indicated in parentheses.

Line Name	Blind test identified the most similar CML entry and their genetic similarity				
	re-generation 1	re-generation 2	re-generation 3	re-generation 4	re-generation 5
CML100	CML100(0.90)	CML100(0.95)	CML100(0.90)	CML100(0.94)	CML100(0.83)
CML106	CML106(1.00)	CML106(1.00)	CML106(1.00)	CML106(1.00)	CML106(1.00)
CML110	CML110(0.90)	CML110(0.90)	CML110(0.90)	CML110(0.90)	CML110(0.90)
CML126	CML126(0.94)	CML126(1.00)	CML126(1.00)	CML126(1.00)	-
CML131	CML131(1.00)	CML131(1.00)	CML131(1.00)	CML131(1.00)	CML131(1.00)
CML136	CML136(1.00)	CML136(1.00)	CML136(1.00)	CML136(1.00)	-
CML14	CML14(0.89)	CML14(0.89)	CML14(0.89)	CML14(0.89)	-
CML17	CML17(0.85)	CML17(0.85)	CML17(0.80)	CML17(0.80)	CML17(0.80)
CML178	CML178(0.93)	CML178(0.93)	CML178(0.92)	CML178(1.00)	CML178(0.86)
CML192	CML192(0.95)	CML192(0.95)	CML192(1.00)	CML192(0.94)	CML192(1.00)
CML193	CML193(0.95)	CML193(0.94)	CML193(0.80)	-	-
CML197	CML197(1.00)	CML197(1.00)	CML197(1.00)	CML197(1.00)	CML197(1.00)
CML233	CML233(1.00)	*CML132(1*.00)*	CML233(1.00)	-	-
CML236	CML236(1.00)	CML236(0.89)	CML236(0.93)	-	-
CML280	CML280(0.95)	CML280(0.95)	CML280(0.95)	CML280(0.90)	-
CML327	CML327(0.90)	CML327(1.00)	CML327(1.00)	CML327(0.89)	CML327(1.00)
CML362	CML362(1.00)	CML362(1.00)	CML362(0.95)	CML362(1.00)	-
CML364	CML364(1.00)	CML364(1.00)	CML364(1.00)	CML364(1.00)	-
CML390	CML390(1.00)	CML390(1.00)	CML390(1.00)	CML390(1.00)	CML390(1.00)
CML393	CML393(1.00)	CML393(1.00)	CML393(1.00)	CML393(1.00)	CML393(1.00)
CML435	CML435(1.00)	CML435(1.00)	CML435(1.00)	CML435(1.00)	-
CML82	CML82(0.94)	CML82(0.94)	CML82(0.94)	CML82(0.94)	-

* The re-generation 2 of CML233 is most similar to a different line.

### Sample number needed for QC

**Fig 6:**
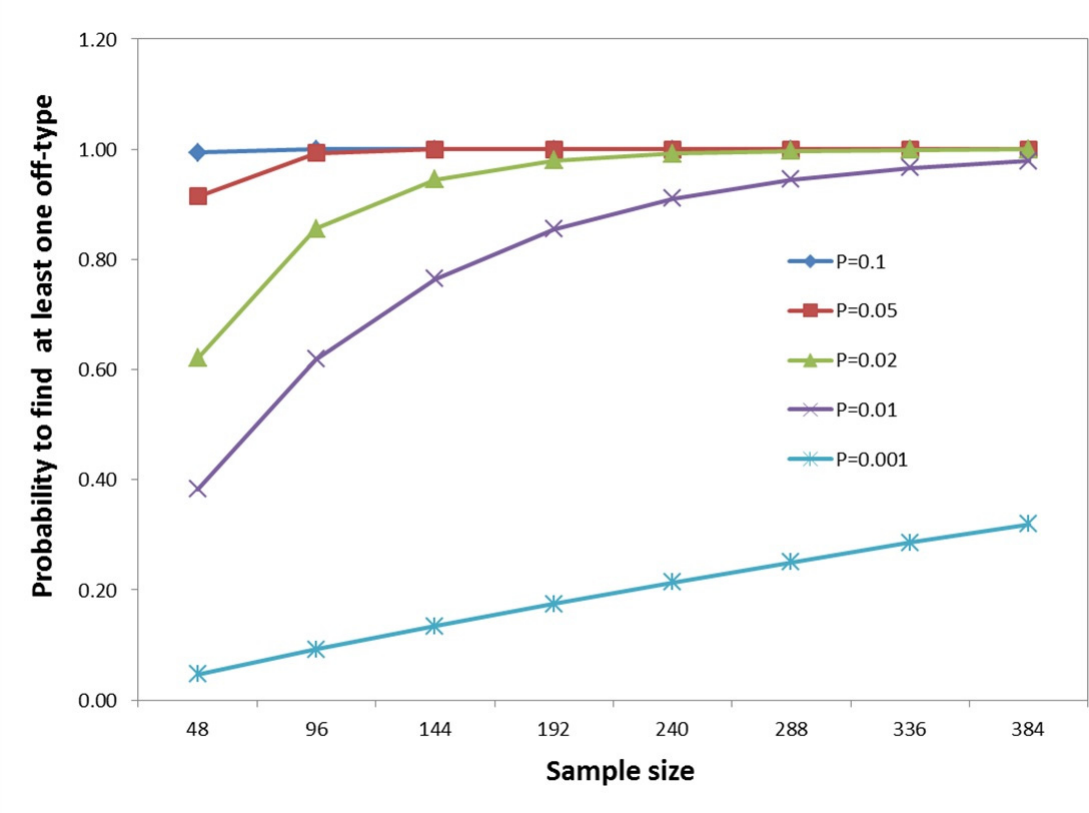
Probability of detecting at least one off-type using different sample sizes at different assumed off-type levels (P) within populations.

The number of individuals sampled from a discrete entity is a key factor for implementation of routine QC genotyping, as it directly influences the cost, time and accuracy of detection of off-types within the entity. The influence of sample number was evaluated using different off-type levels (0.001, 0.01, 0.02, 0.05 and 0.1) assuming the marker detection power was 100% (Fig 6). As anticipated, the probability of detecting off-types increases with increasing sample size. For example, at 0.01 population contamination level, the off-type detection rate rose from around 40% to 97.8% as the sample number increased from 48 to 384. If the allowed off-type composition of a sample was 5%, ninety six samples per population would provide close to 100% probability of detection of off-types at this level. At an allowed 1% population off-type, a sample size of 384 individuals would be required to have a similar detection probability. A level of off-type detection of 2% is similar to the error rate for genotyping in many platforms and would offer the most stringent level for QC for inbreeding programs and genebank applications. Using 192 individuals per entry for QC genotyping would result in an off-type detection chance close to 98% (Fig 6). To determine the optimal sample size, we can look at the capacity to detect contamination in populations with defined percentages of off-types. Considering different number of off-types were detected in the sample from 0 to 3 (S7 Fig), the upper limit with a probability of 0.95 was approximately 2% when there was one off-type in a sample of 192 entities. It showed if two samples were detected as being different in a set of 96, the whole entry would have an upper limit of 5%. If two off-types were detected in sample of 192 individuals, the whole entry would have an upper limit of 2.5% contamination with probability of 0.95.

## Discussion

The main purpose of routine QC genotyping is to identify contamination or mislabeling of germplasm during regeneration, seed increase or seed distribution. In order to achieve this, a balance needs to be maintained between accuracy of detection and efficiency (both in terms of cost and time). It is often assumed that more markers result in higher accuracy, this accuracy being achieved at a higher cost. Optimization of the balance between accuracy and cost is the main concern of the QC marker selection. Semagn et al. [14] suggested using 50–100 single plex assay SNPs for QC genotyping at a cost estimated at 7-15USD per individual. The effectiveness of this approach in differentiating germplasm entries genotypically was confirmed by Ertiro et al. [25], in a study employing 191 KASP and 257,268 GbS markers. Selection of markers to use in QC analysis is important independent of genotyping platform. Use of the best markers to separate germplasm is desired across any platform; informative data generation is a fundamental pre-requisite. In single plex marker systems cost and time for data generation scales with increased marker numbers. In contrast, in most sequencing based systems where marker number is largely independent of cost up to tens to hundreds of thousands of markers, the higher levels of missing data can result in erroneous interpretation, so sub-selection from the many thousands of markers generated for those markers with high and repeatable representation across samples is desired. In this paper, we propose the use of two separate sets of markers to conduct QC, each focusing on different types of QC. The first was a broad QC focusing on identity of a sample. This employed a minimum of 80 carefully selected markers to distinguish each of the CML entries from one another. It is important to conduct this type of QC before starting new breeding crosses, to ensure the identity and purity of the founding parents and to evaluate the levels of residual heterogeneity within them. The second approach was rapid QC for seed production. This approach used a smaller sub-set of only ten selected markers. Application of the rapid QC was proposed to quickly assess mislabeling of entries across the entire panel of CMLs, achieving a 99% chance of detection. Choosing from the two different QC approaches, dependent upon the specific application, will ultimately improve the effectiveness of QC and can lower genotyping costs and data turnaround times within specific platforms.

Sample number is also a key factor for QC genotyping because it directly influences the cost, time and accuracy of detection of off-types. One hundred individuals have been used for hybrid purity testing [44]. In this paper we calculated the detection probability based on assuming a known off-types rate and 100% marker detection efficiency (Fig 6). A tolerable level of off-type detection of 2% is generally suitable for most breeding programs and genebanks, indeed this is close to the error rate of many genotyping platforms. Using this level of detection, sampling 192 individuals from each CML entry used for QC genotype would result in an off-type detection probability of close to 98%. Using this detection system, if two off-types were detected in the sub-set of 192 individuals, the upper limit of off-types at 0.95 probabilities would be 2.5%. Based on this analysis, we suggest taking a minimum of 192 samples from each line submitted for QC genotyping. A threshold could be set at two off-types per entry, signifying that if two or fewer off-types were detected per entry, it passes the QC test. Otherwise, the entry would need to be retested and if failing a second time regenerated again, possibly using a different parental seed source.

There are a number of widely used SNP genotyping platforms, ranging from single SNP methods such as KASP™ from LGC Genomics [23] (http://www.lgcgroup.com) and TaqMan™ from Applied Biosystems [45] (http://www.lifetechnologies.com), to larger fixed array based systems, including BeadXpress™ and GoldenGate™ from Illumina [28,46] (http://www.illumina.com/) and Axiom® Genotyping Array from Affymetrixs (http://www.affymetrix.com), and finally to the next-generation sequence based genotyping platforms, such as KeyGene® SNPSelect (http://www.keygene.com) GbS from Cornell and DArTSeq, developed by Diversity Arrays Technology (http://www.diversityarrays.com) [18,47]. The absolute cost per marker data point is on average higher with single plex systems, however the total cost of operation per sample is lower when only a limited number of markers are required. The lower cost per sample in the low marker number range is not simply influenced by assay cost but also by the DNA extraction costs- single plex systems are generally the most tolerant of low-cost, low purity DNA extraction methods. The large marker numbers currently generated by some of the chip and sequence based genotyping platforms are more suited to application in discovery work, such as GWAS, and some breeding implementations, such as genomic selection. The number of markers generated by these systems is “overkill” for QC work and can offer a prohibitively high per sample cost with currently available technology packages when samples from individual plants are considered.

Here we have based our analysis on the use of the lowest number of markers to employ in QC genotyping to enable generation or selection of markers across multiple platforms. The analysis framework used to select markers is applicable across the genotyping system(s) used in analysis. The cost-benefit ratio may well swing in favor of sequence- and chip-based platforms and away from single plex systems in the near future as; the ability to increasingly multiplex in sequence and array-based platforms improves, the ability to better define specific sets of markers in sequence based systems (e.g. DArTcap) is commercialized by service providers and as the potential to use composite sample based genotyping to assess homogeneity is further realized. A summary of the current genotyping approaches used at CIMMYT, the approaches being reviewed and cost analysis is presented in S4 Table.

The stage at which QC genotyping is conducted is also a critical consideration. A wide range of potential intervention points for QC genotyping are available during the breeding and conservation cycles, including vegetative plants in the field prior to pollination, seed or seedlings after harvest, and seed or seedlings prior to distribution. Each offers a different insight into the genetic status of germplasm. In the broader context, when sampling seed directly, one needs to consider the potential confounding effects of polyploid endosperm in non-homozygous germplasm. Different genotyping platforms and SNP markers respond in different ways to increased maternal effect (for example, the definition of clear homozygote and heterozygote boundaries can be influenced by 3n samples) and detection of contamination can be confounded by this in some cases (author personal experience). Careful empirical assessment of this is required if using seed derived DNA across the different genotyping platforms available.

In conclusion, the work presented here outlines the framework and output for the selection of the minimum number of high quality, robust markers for two complimentary QC genotyping approaches for maize lines. The methods and analysis presented offer a roadmap for the selection of markers for other germplasm resources while providing some reflection on the consideration of genotyping platforms.

## Supporting Information

S1 Dataset The CurlyWhirly formatted file of the Principal Coordinate Analysis (PCoA) results with mega environments and grain color classification. (TXT)

S2 Dataset Optimal marker subsets for broad (S1666) and rapid QC (QC114). (TXT)

S3 Dataset The genotypic similarity of the 22 original CMLs used for QC marker validation. (XLSX)

S1 Fig Principal Coordinate Analysis (PCoA) for all CMLs with mega environment classification. The 3-D video of this figure can be found in “S1 Video” and the original dataset for the figure and video is available in “S1 Dataset” which can be opened by CurlyWhirly software (https://ics.hutton.ac.uk/curlywhirly/download-curlywhirly/).
(TIF)

S2 Fig Principal Coordinate Analysis (PCoA) for all CMLs with grain colour classification. The 3-D video of this figure can be found in “S2 Video” and the original dataset for the figure and video is available in “S1 Dataset” which can be opened by CurlyWhirly software (https://ics.hutton.ac.uk/curlywhirly/download-curlywhirly/).
(TIF)

S3 Fig Hierarchical Clustering showing all of the CMLs with germplasm source classification. The CIMMYT Breeding Program and the source population are indicated for each cluster. In the source materials, “P” indicates the CIMMYT synthetic population founder; “G” indicates the synthetic gene pool founder. MA/ST = mid-altitude/sub-tropical adaptation; MBRET = multiple borer resistance and *Exserohilum turcicum* resistant fonder; SintAmTSR = synthetic yellow and tar spot complex resistant founder, SA = acid soil tolerance founder. The breeding program and source population information of all CMLs can be found: http://hdl.handle.net/11529/10246.
(TIF)

S4 Fig PCA representation of marker groups defined by PCA and K-means methods. (TIF)

S5 Fig Influence of marker sequencing coverage on marker quality parameters. Heterogeneity, missing values and number of markers are shown.
(TIF)

S6 Fig Influence of marker numbers on CML separation evaluated using broad QC maker set S1666 to estimate the number of markers needed for rapid QC. (TIF)

S7 Fig Estimation of the percentage of off-type samples within a population, at percentile 0.95 of the posterior distribution, from sub-population sampling using different sample size and different levels of off-type detection. (TIF)

S1 Table The number of regenerations and their source of the 22 multiple regeneration CMLs used in present study. (DOCX)

S2 Table Influence of marker filter parameters on the number of SNP markers defined from the total dataset for QC analysis. (DOCX)

S3 Table List of trait-converted CMLs, corresponding recurrent parents and introgression traits. (DOCX)

S4 Table Summary of the current genotyping approaches, approximate costs and informatics needs assessed experienced by CIMMYT. (DOCX)

S1 Video Principal Coordinate Analysis (PCoA) for all CMLs with mega environment classification. (AVI)

S2 Video Principal Coordinate Analysis (PCoA) for all CMLs with grain color classification. (AVI)
