# Supplementary figures and images for: The Development of Quality Control Genotyping Approaches: A Case Study Using Elite Maize Lines
Source: PLoS One. 2016 Jun 9;11(6):e0157236. doi: 10.1371/journal.pone.0157236 (PMC4900658; doi:10.1371/journal.pone.0157236)

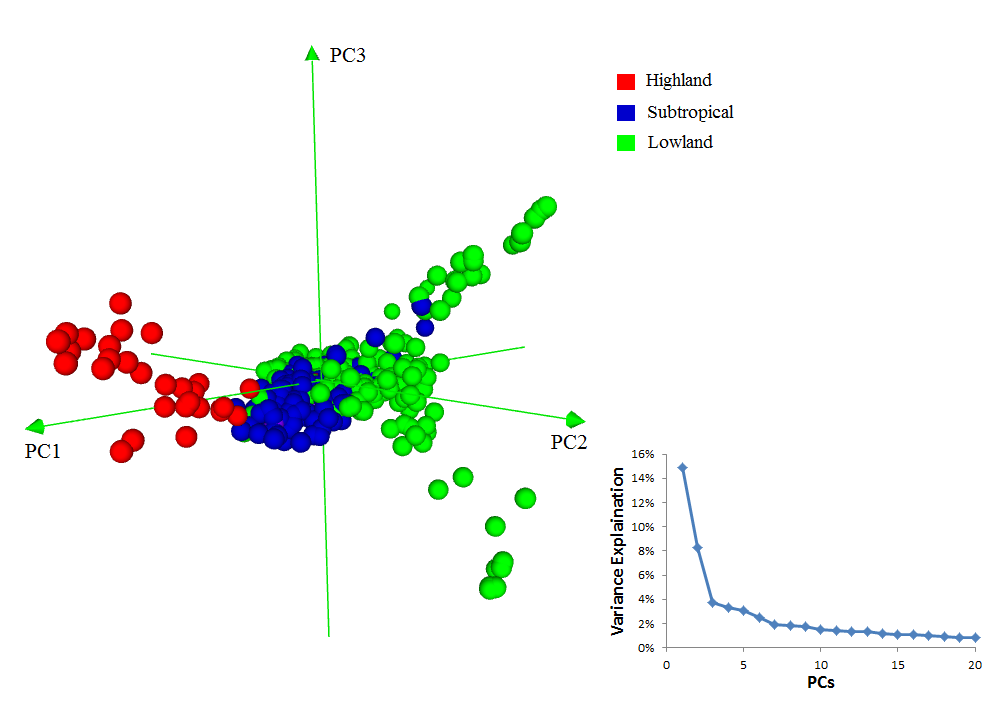

Supplement: S1 Fig — The 3-D video of this figure can be found in “S1 Video” and the original dataset for the figure and video is available in “S1 Dataset” which can be opened by CurlyWhirly software (https://ics.hutton.ac.uk/curlywhirly/download-curlywhirly/). (TIF) [file pone.0157236.s004.tif]

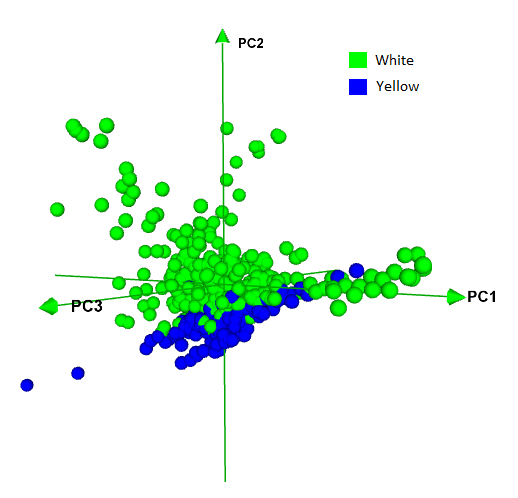

Supplement: S2 Fig — The 3-D video of this figure can be found in “S2 Video” and the original dataset for the figure and video is available in “S1 Dataset” which can be opened by CurlyWhirly software (https://ics.hutton.ac.uk/curlywhirly/download-curlywhirly/). (TIF) [file pone.0157236.s005.tif]

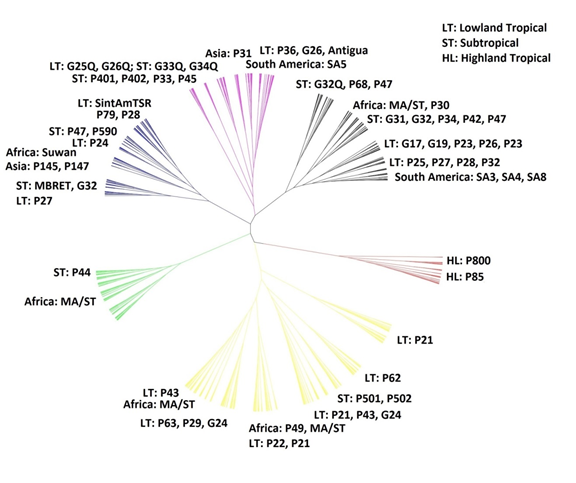

Supplement: S3 Fig — The CIMMYT Breeding Program and the source population are indicated for each cluster. In the source materials, “P” indicates the CIMMYT synthetic population founder; “G” indicates the synthetic gene pool founder. MA/ST = mid-altitude/sub-tropical adaptation; MBRET = multiple borer resistance and Exserohilum turcicum resistant fonder; SintAmTSR = synthetic yellow and tar spot complex resistant founder, SA = acid soil tolerance founder. The breeding program and source population information of all CMLs can be found: http://hdl.handle.net/11529/10246. (TIF) [file pone.0157236.s006.tif]

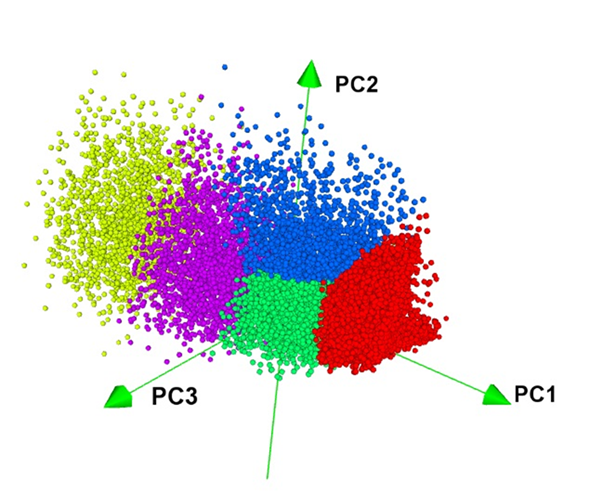

Supplement: S4 Fig — (TIF) [file pone.0157236.s007.tif]

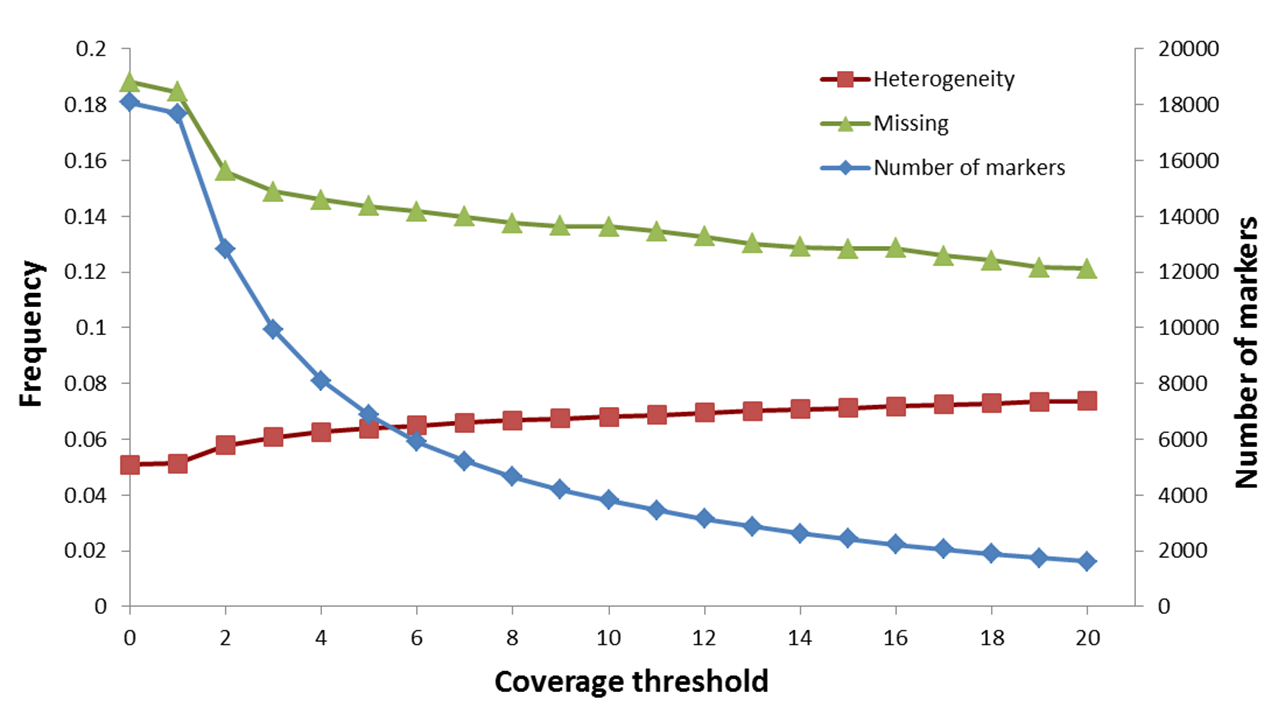

Supplement: S5 Fig — Heterogeneity, missing values and number of markers are shown. (TIF) [file pone.0157236.s008.tif]

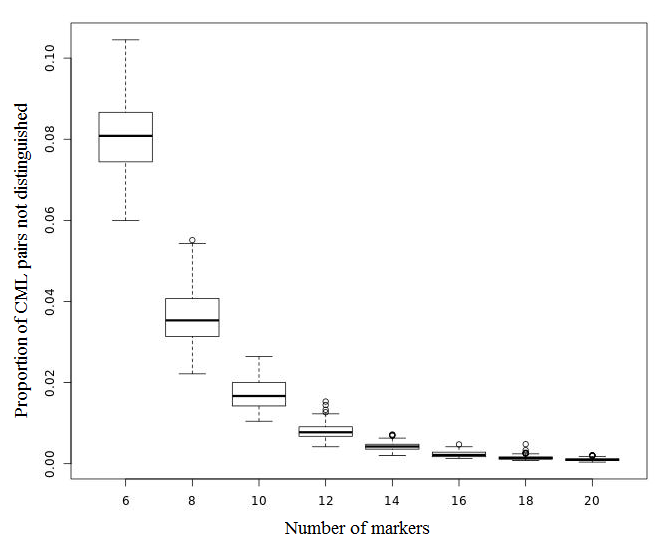

Supplement: S6 Fig — (TIF) [file pone.0157236.s009.tif]

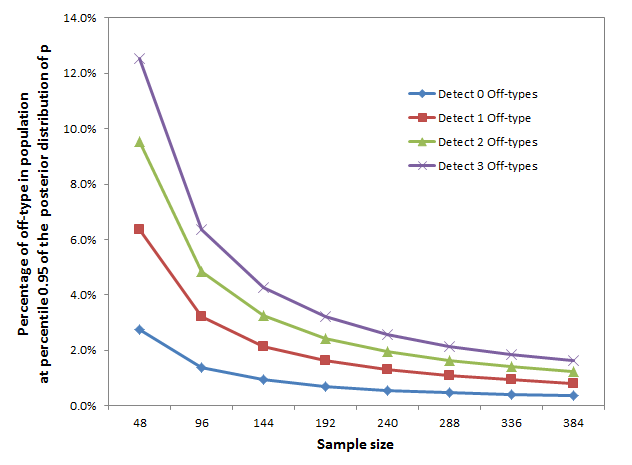

Supplement: S7 Fig — (TIF) [file pone.0157236.s010.tif]
