# Supplementary material for: The Development of Quality Control Genotyping Approaches: A Case Study Using Elite Maize Lines
Source: PLoS One. 2016 Jun 9;11(6):e0157236. doi: 10.1371/journal.pone.0157236 (PMC4900658; doi:10.1371/journal.pone.0157236)
Supplement: S1 Table — (DOCX) [file pone.0157236.s011.docx]

**S1 Table. The number of regenerations and their source of 22 multiple regeneration CMLs used in present study**

| Name | Number of | Source of the re-generation | | | | |
| --- | --- | --- | --- | --- | --- | --- |
|  | Re-generation | re-generation 1 | re-generation 2 | re-generation 3 | re-generation 4 | re-generation 5 |
| CML100 | 5 | AF09A-0903-36 | TL08A-1903-121 | TL08B-6903-44 | AF09A-0903-36 | TL07A-1903-275 |
| CML106 | 5 | TL05B-6903-140 | TL07A-1903-277 | TL08B-6903-17 | TL07A-1903-277 | TL08A-1903-11 |
| CML110 | 5 | TL06B-6903-119 | TL06B-6903-119 | TL07A-1903-279 | TL08A-1903-96 | TL09B-6903-20 |
| CML126 | 4 | TL06B-6903-135 | TL08A-1903-122 | AF09A-0903-37 | TL08B-6903-45 | - |
| CML131 | 5 | TL06B-6903-140 | TL06B-6903-140 | TL08B-6903-31 | TL08A-1903-75 | TL07A-1903-131 |
| CML136 | 4 | TL06B-6903-145 | BA10-2903-159 | TL07A-1903-290 | TL08A-1903-78 | - |
| CML14 | 4 | AF11A-0903-10 | TL07A-1903-14 | AF11B-5903-45 | AF08A-0903-3 | - |
| CML17 | 5 | AF11A-0903-22 | AF11A-0903-22 | AF08A-0903-59 | AF06B-5903-17 | AF07A-0903-148 |
| CML178 | 5 | TL06B-6903-154 | TL06B-6903-154 | TL08A-1903-182 | TL08B-6903-57 | AF10A-0903-8 |
| CML192 | 5 | TL06B-6903-168 | AF09A-0903-39 | TL07A-1903-298 | TL09B-6903-27 | TL06B-6903-168 |
| CML193 | 3 | BA10-2903-172 | AF09A-0903-40 | TL07A-1903-119 | - | - |
| CML197 | 5 | AF10A-0903-26 | TL11B-6903-158 | AF10A-0903-26 | AF08A-0903-98 | TL06B-6903-194 |
| CML233 | 3 | TL95B-6294-44 | AF09A-0903-1 | AF08A-0903-14 | - | - |
| CML236 | 3 | TL10B-6903-115 | TL10B-6903-115 | AF07A-0903-85 | - | - |
| CML280 | 4 | AF11A-0903-20 | AF11B-5903-57 | AF11A-0903-20 | AF08A-0903-183 | - |
| CML327 | 5 | TL08B-6903-24 | TL06B-6903-199 | TL07A-1903-382 | TL08A-1903-46 | TL09B-6903-37 |
| CML362 | 4 | AF09A-0903-23 | AF09A-0903-23 | AF10A-0903-13 | TL07A-1903-158 | - |
| CML364 | 4 | AF10A-0903-14 | TL09B-6903-41 | AF10A-0903-14 | TL08B-6903-30 | - |
| CML390 | 5 | AF09A-0903-2 | AF09A-0903-2 | AF08A-0903-17 | TL11B-6903-155 | TL07A-1903-183 |
| CML393 | 5 | BA10-2903-169 | TL09B-6903-42 | AF10A-0903-18 | TL07A-1903-186 | TL08B-6903-4 |
| CML435 | 4 | TL11A-1903-4 | TL11A-1903-4 | TL06B-6903-184 | TL07A-1903-378 | - |
| CML82 | 4 | AF10A-0903-2 | TL05A-1903-215 | TL09B-6903-13 | TL11B-6903-138 | - |
