# Supplementary material for: The Development of Quality Control Genotyping Approaches: A Case Study Using Elite Maize Lines
Source: PLoS One. 2016 Jun 9;11(6):e0157236. doi: 10.1371/journal.pone.0157236 (PMC4900658; doi:10.1371/journal.pone.0157236)
Supplement: S2 Table — (DOCX) [file pone.0157236.s012.docx]

**S2 Table. Influence of marker filter parameters on the number of SNP markers defined from the total dataset for QC analysis.**

| Marker Filter Parameter | Marker Number |
| --- | --- |
| Total Marker | 88,600 |
| Missing Proportion > 0.4 | 23,975 |
| Marker Minor Allele Frequency < 0.05 | 45,215 |
| Marker Heterozygosity > 0.1 | 15,837 |
| Final Markers used for future analysis | 18,082 |
