# Supplementary material for: The Development of Quality Control Genotyping Approaches: A Case Study Using Elite Maize Lines
Source: PLoS One. 2016 Jun 9;11(6):e0157236. doi: 10.1371/journal.pone.0157236 (PMC4900658; doi:10.1371/journal.pone.0157236)
Supplement: S3 Table — (DOCX) [file pone.0157236.s013.docx]

**S3 Table. List of trait-converted CMLs, corresponding recurrent parents and introgression traits.**

| Converted Line | Recurrent Parent | Introgression Trait |
| --- | --- | --- |
| CML503 | CML264 | QPM |
| CML524 | CML242 | QPM |
| CML525 | CML244 | QPM |
| CML526 | CML246 | QPM |
| CML527 | CML349 | QPM |
| CML528 | CML352 | QPM |
| CML529 | CML354 | QPM |
| CML512 | CML78 | IR |
| CML513 | CML202 | IR |
| CML514 | CML204 | IR |
| CML515 | CML247 | IR |
| CML516 | CML254 | IR |
| CML517 | CML312 | IR |
| CML518 | CML373 | IR |
| CML519 | CML384 | IR |
| CML520 | CML390 | IR |
| CML521 | CML395 | IR |
| CML522 | CML444 | IR |
| CML523 | CML445 | IR |

Note: QPM: quality protein maize controlled by the o2 gene; IR: native imidazolinone resistance controlled by the als2 gene.
