# Supplementary material for: The Development of Quality Control Genotyping Approaches: A Case Study Using Elite Maize Lines
Source: PLoS One. 2016 Jun 9;11(6):e0157236. doi: 10.1371/journal.pone.0157236 (PMC4900658; doi:10.1371/journal.pone.0157236)
Supplement: S4 Table — (DOCX) [file pone.0157236.s014.docx]

**S4 Table. Summary of the current genotyping approaches, approximate costs and informatics needs assessed experienced by CIMMYT.**

| Genotyping service system | Number of SNP markers | SNP marker repeatability across samples | Example cost per sample^a^ | Heterozygote calling | Informatics needs | Experienced data turnaround time^a^ |
| --- | --- | --- | --- | --- | --- | --- |
| GbS | 800,000+ | Low-Moderate | $30 | No, imputation required | Extensive calling pipeline and advanced data handling and analysis system | Minimum 28 days |
| DArTSeq | 100-350,000 | Moderate | $45 | Yes | Advanced data handling and analysis system | Minimum 10 days |
| KASP | 1 + | High | $3 for 10 SNP  $8 for 80 SNP | Yes | Basic data handling and analysis system | 21 days^c^ |
| DArTcap^b^ | 1 - 500 | High | $7 | Yes | Basic data handling and analysis system | Minimum 10 days |

^a^: Calculation was based on 96 samples. ^b^: Pre-selection of fragments. ^c^: lower turnaround times available at higher per sample pricing.
